# Elicitin-Induced Distal Systemic Resistance in Plants is Mediated Through the Protein–Protein Interactions Influenced by Selected Lysine Residues

**DOI:** 10.3389/fpls.2016.00059

**Published:** 2016-02-05

**Authors:** Hana Uhlíková, Michal Obořil, Jitka Klempová, Ondrej Šedo, Zbyněk Zdráhal, Tomáš Kašparovský, Petr Skládal, Jan Lochman

**Affiliations:** ^1^Department of Biochemistry, Faculty of Science, Masaryk UniversityBrno, Czech Republic; ^2^Research Group Proteomics, Central European Institute of Technology, Masaryk UniversityBrno, Czech Republic; ^3^National Centre for Biomolecular Research, Faculty of Science, Masaryk UniversityBrno, Czech Republic

**Keywords:** cryptogein, lysine residues, resistance, movement, dimerization, lipid transfer proteins

## Abstract

Elicitins are a family of small proteins with sterol-binding activity that are secreted by *Phytophthora* and *Pythium* sp. classified as oomycete PAMPs. Although α- and β-elicitins bind with the same affinity to one high affinity binding site on the plasma membrane, β-elicitins (possessing 6–7 lysine residues) are generally 50- to 100-fold more active at inducing distal HR and systemic resistance than the α-isoforms (with only 1–3 lysine residues). To examine the role of lysine residues in elicitin biological activity, we employed site-directed mutagenesis to prepare a series of β-elicitin cryptogein variants with mutations on specific lysine residues. In contrast to direct infiltration of protein into leaves, application to the stem revealed a rough correlation between protein’s charge and biological activity, resulting in protection against *Phytophthora parasitica*. A detailed analysis of proteins’ movement in plants showed no substantial differences in distribution through phloem indicating differences in consequent apoplastic or symplastic transport. In this process, an important role of homodimer formation together with the ability to form a heterodimer with potential partner represented by endogenous plants LTPs is suggested. Our work demonstrates a key role of selected lysine residues in these interactions and stresses the importance of processes preceding elicitin recognition responsible for induction of distal systemic resistance.

## Introduction

Plants have developed a complex innate immune system to prevent the spread of pathogens and provide protection. So far, two main branches of the plant immune system have been identified: pathogen-associated molecular patterns (PAMPs)-triggered immunity (PTI) and effector-triggered immunity (ETI) (Nürnberger et al., 2004; Zipfel et al., 2004; Jones and Dangl, 2006; Boller and Felix, 2009).

Elicitins are among the most well-known oomycetes PAMPs. They are highly conserved sterol-binding proteins secreted by *Phytophthora* and *Pythium* sp. which have been shown to induce the hypersensitive response (HR) in several plants, such as *Nicotiana* species and some radish and rape cultivars (Ricci et al., 1989; Kamoun et al., 1993; Panabieres et al., 1995; Ponchet et al., 1999). Almost all known elicitins contain a 98 amino acid domain that lacks tryptophan, histidine and arginine residues but has six cysteine residues in conserved positions, forming three structurally determinant disulfide bridges (Boissy et al., 1999; Rodrigues et al., 2006). Based on the primary structure of elicitins, five different classes have been identified, where elicitins in class I only contain the elicitin domain of 98 amino acids (Kamoun et al., 1993, 1997; Ponchet et al., 1999). Class I elicitins can be further separated according to their pI, i.e., as either acidic (α, pI < 5) or basic (β, pI > 7.5). Both forms can be produced within the same *Phytophthora* species. However, compared to α-elicitins, β-elicitins are secreted by a restricted range of species and appear to be ancestors of other elicitins (Ponchet et al., 1999).

Most of the previous work on elicitins has been carried out on tobacco plants. In general, two basic methods of application have been employed: either application on the stem of decapitated plants or direct infiltration into leaf mesophyll. The first mode of treatment leads to the systemic movement of acidic and basic elicitins as well as pythins (an elicitin-like protein produced by *Pythium*) across the plant (Devergne et al., 1992; Keller et al., 1996; Picard et al., 2000), but only basic elicitins induce a distal HR and systemic resistance against pathogens. In the latter mode, acidic and basic elicitins induce a HR, however, they are restricted to the infiltration site without any movement to surrounding areas, inducing only local acquired resistance (LAR) against the pathogens (Dorey et al., 1997). Thus not movement of elicitins in vascular system is absolutely necessary for their ability to induce systemic resistance in plants (Keller et al., 1996) but the other factors related to elicitins transport to parenchyma cells could play an important role in their activity. Although α- and β-elicitins have been shown to bind with equivalent affinity to the same high affinity binding site on the plasma membrane (Bourque et al., 1998), one important difference was detected; after application on decapitated tobacco plants, β-elicitins were shown to be 50- to 100-fold more active in inducing a distal HR and systemic acquired resistance (SAR) than α -isoforms. Moreover, Bourque et al. (1998) showed that β-elicitins in a tobacco cell suspension are at least 10-times more efficient at inducing extracellular pH changes, active oxygen species (AOS) production or Ca^2+^ influx compared to α-elicitins. Recently, in wild potato *Solanum microdontum* a receptor-like protein ELR (elicitin response) mediating extracellular recognition of the elicitin domain was demonstrated, although the binding to elicitins still needs to be demonstrated (Du et al., 2015). Even though elicitin binding seems to be a prerequisite for the induction of the plant defense response, like the AVR9/Cf-9 interaction in tomato or NIP1/Rrs1 in barley, an effective response is only observed in the presence of a third interacting component (Bourque et al., 1999; van’t Slot et al., 2007; Wulff et al., 2009). Kanzaki et al. (2008) showed that elicitin INF1 could interact with the intracellular kinase domain of NbLRK1 kinase. Although at first glance their results suggesting the intracellular recognition of elicitins seem to be enigmatic, they fully correspond with the measured stimulation of clathrin-mediated endocytosis by the elicitin cryptogein in tobacco cells or localization of the elicitin quercinin inside cells of host oak plants by immunocytology (Brummer et al., 2002; Leborgne-Castel et al., 2008). Finally, ligand-induced receptor endocytosis has been suggested to be involved in the activation of plant defense mechanisms (Robatzek, 2007).

Based on recent results, the activity of elicitins is probably dependent on the presence of specific residues, the most likely candidates being the lysine residues in the A and D helices of basic elicitins (Dokladal et al., 2012). This assumption is supported by the observed correlation between necrotic index and pI (Pernollet et al., 1993) and clear impact of the Lys13Val mutation in helix A on the induction of a defense response in tobacco plants (Pleskova et al., 2011).

The main goal of the present study was to investigate the role of individual Lys residues responsible for the global charge of elicitins on the ability to induce distal systemic resistance. As a model, we used the very efficient basic elicitin cryptogein containing six Lys residues secreted by *Phytophthora cryptogea* and tobacco plants. Using site-directed mutagenesis, five Lys residues were systematically replaced by Thr residues and the influence of the mutations on the defense reaction in tobacco plants was determined with respect to (i) changes in the biochemical properties, (ii) activation of resistance to the pathogen *P. parasitica*, and (iii) movement in plants.

## Materials and Methods

### Plant Material

Tobacco seeds (*Nicotiana tabacum* L. cv. *Xanthi*) were sown in peat soil. Once germinated, the plants were grown under controlled conditions (23°C, 16 h light, 6000 lux, 75% r.h.). The experiments were carried out on 8-week-old plants.

### Resistance Analysis

Systemic acquired resistance was induced by elicitin application (Ponchet et al., 1999): plants were decapitated and their stems treated with 20 μl of water or 5 μM aqueous solutions of proteins. After 48 h inoculations of *P. parasitica* were performed by infiltrating parenchyma tissue of non-necrotic parts of leaves with a 50 μl suspension containing 100 zoospores (Hugot et al., 1999). In each experiment, at least four consecutive leaves received two infiltrations of zoospore suspension each. Susceptibility and resistance were evaluated by measuring the area over which disease symptoms were observed on each leaf at different times after inoculation because the development of disease symptoms directly correlates with the development of the *oomycete* (Galiana et al., 1997). All experiments were performed at least three times with three replicate plants. Results were presented as mean ± standard deviation. Student’s *t*-test was used to analyze differences between two groups.

### Expression and Purification of Recombinant Proteins

The X24 gene from *P. cryptogea* with α-secretion factor (*Saccharomyces cerevisiae*) was inserted into a pPIC9 vector (Life Technologies, USA). In addition, a glycine residue was added on the N-terminus of the recombinant protein in order to improve the processing ability of the KEX2 protease (α-secretion factor cleavage). A QuikChange kit (Stratagene, France) for direct mutagenesis was then used to construct the desired expression vectors by using oligonucleotides listed in Supplementary Table S1. All vectors were validated by DNA sequencing. The constructed vectors were transformed into *Pichia pastoris* strain GS115. Screening for optimal protein production was performed and the most suitable strain was cultivated in a Biostat B-DCU bioreactor (Sartorius, Germany) using a previously described protocol (Wood and Komives, 1999). The expressed proteins were purified by ultrafiltration and Fast Protein Liquid Chromatography using a Source S15 column as described before (Pleskova et al., 2011). The molecular weights (MW) of the purified proteins were determined by MALDI-TOF spectroscopy.

### Chemical Crosslinking of Proteins

The EGS (ethylene glycol bis[succinimidylsuccinate]; Sigma, Germany) crosslinking reagent containing amine-reactive NHS-ester reacting ends around a 12-atom spacer arm was used. Twenty microliters of 10 μM solutions of individual proteins was crosslinked according to manufacture instruction and reaction was stopped by addition of TRIS buffer. In case of wt cryptogein dimer preparation 1 mg of protein was crosslinked and dimer was consequently purified on HP 1100 High-Performance Liquid Chromatography system (Agilent, Germany) equipped with a DAD detector set at 280 nm, an Discovery Bio Wide Pore C-5 (10 mm I.D. × 5 cm) reverse-phase column (Supelco) as the stationary phase and solution A (100 mM formiate, 50% ACN) and Solution B (20 mM formiate, 10 mM ammonium sulfate, 20% ACN) as the mobile phases. The elution gradient was as follows: 0 5 min, 0 5% of B; 5 15 min, 5 15% of B; 15 20 min, 15 20% of B; 20 21 min, 20 60% of B. The flow rate was 1 ml min^-1^ and the column temperature was 23°C. The MW of the purified protein was determined by MALDI-TOF spectroscopy.

### MALDI MS Analyses

MALDI-TOF mass spectrometric analysis was carried out using and Ultraflextreme instrument (Bruker Daltonik, Bremen, Germany) with alpha-cyano-4-hydroxycinnamic acid as the MALDI matrix. The Flex Analysis 3.0 and MS Biotools 3.1 (Bruker Daltonik) software were used for data processing.

### Thermal Shift Assay

Thermal shift assays (TSAs) were performed using a LightCycler 480 instrument (Roche) and the environmentally sensitive dye SYPRO orange (Invitrogen). Thermal manipulation and fluorescence dye detection were carried out according to a previously published method (Nettleship et al., 2008). Optimal assay conditions for cryptogein, i.e., 50 mM MES buffer (pH = 5.5) with 25 mM KI and 12.5x SYPRO Orange dye, were used in all assays. Custom filter configurations were used corresponding to the optimal excitation (492 nm) and emission (610 nm) wavelengths for SYPRO orange dye. The temperature was increased by 0.1°C/s from 25–95°C and all experiments were analyzed in triplicate. Assay reactions were performed in 96-well white PCR plates (Roche). All the reported *T*_m_ values were calculated from a sigmoidal analysis of the raw fluorescence data.

### Sterol Transfer Assay

The sterol transfer assay was based on the self-quenching properties of dehydroergosterol (DHE; EX/EM = 325/375 nm). In 2-(*N*-morpholino)ethanesulfonic acid (MES) buffer, two types of micelles are formed progressively: acceptor (stigmasterol) micelles and donor (dehydroergosterol) micelles. Thus, each of the studied proteins was individually added to a stirred mixture of micelles and then a time-dependent fluorescence curve was recorded (Mikes et al., 1998). The DHE transfer activity of individual proteins was evaluated using a computer program written in Python 2.7, which performed numerical integration of the time-dependent fluorescence curves against the straight line obtained by linear regression of the baseline according to the following equation:

(1) $$A_{t}=\int_{t_{a}}^{t_{e}}F(t)dt-\int_{t_{a}}^{t_{e}}(at+b)dt$$

where

*A*_t_ is the calculated transfer ability of the studied protein in relative fluorescence units (RFU).

*t*_a_ is the time of addition of studied protein in seconds.

*t*_e_ is the time at the end of the evaluation interval in seconds.

*F*(t) is the time-dependent fluorescence curve in RFU.

*a* and *b* are parameters of the straight line obtained by linear regression of the baseline.

*t* is the time from start of data collection seconds.

Finally, the transfer ability of each protein was compared to that of wild-type (wt) cryptogein after subtraction of a negative control represented by aprotinin.

### Transcription Levels of Defense Genes

The expression of genes in leaf tissues was analyzed by real-time quantitative PCR (RT-qPCR) using the fluorescent intercalating dye SYBR-Green and a Light Cycler 480 (Roche). Total RNA was isolated from 100 mg of leaf tissue using TRI reagent (Ambion, USA) and purified using the TURBO DNA-free kit (Ambion, USA). Reverse transcriptase reactions were performed in a ImProm-II reverse transcription system (Promega, USA) with 0.4 μg of total RNA in a volume of 20 μl according to the manufacturer’s instructions. cDNA was amplified by qPCR using gene-specific primers (Supplementary Table S1) and GoTaq qPCR Master Mix (Promega, USA) according to the manufacturer’s instructions. PCR amplification was carried out as follows: 45 cycles of DNA denaturation at 95°C for 20 s, annealing and extension at 60°C for 40 s. Three replicates were analyzed for each sample. The transcript level of each gene was normalized to that of elongation factor 1α (EF-1α) to evaluate the gene expression relative to an endogenous control by the ΔΔ*C*_T_ method. Previously, it was shown that the expression of elongation factor 1-α is not influenced by different stress conditions (Nicot et al., 2005).

### Detection of Biotin-Labeled Proteins in Leaves

Selected proteins were biotin labeled using biotinamidohexanoyl-6-aminohexanoic acid N-hydroxysuccinimide ester (B3295 Sigma–Aldrich) according to manufacturer’s instructions for 1 h at room temperature. To three different detached tobacco leaves 125 μl of 750 nM solutions of biotin-labeled proteins were aspirated through petiole or three different tobacco leaves were directly infiltrated with 100 μl of 50 nM biotin-labeled proteins. Samples were collected in indicated times after application and protein contents were extracted by treatment for 1 h at 45°C in a solution containing 50 mM phosphate buffer (pH = 7) with 1 mM EDTA, 1 mM 2-mercaptoethanol and 1% SDS. Isolated proteins were separated by denaturing SDS-PAGE (15% acrylamide) followed by western blot and detected by streptavidin labeled with horseradish peroxidase using Luminata Crescendo Luminescence Substrate (Millipore).

### Preparation of Biosensors and Measuring Procedure

Piezoelectric quartz crystal resonators (10 MHz, optically polished surface, 5 mm gold electrodes) were obtained from International Crystal Manufacturing (ICM, Oklahoma City, OK, USA) and prepared as described previously (Svozilová et al., 2009).

For measurements, a piezoelectric biosensor was mounted in a thin-layer flow-through cell with internal volume of 10 μL. A peristaltic pump (Minipuls MP3, Gilson, Villiers Le Bell, France) was used to provide a flow rate through the thin-layer cell of 40 μL/min. The piezoelectric crystal was driven by a Lever oscillator (ICM) and counter UZ 2400 (Grundig, Fuerth, Germany) connected to a computer. A custom written LabTools program was used to display and record the frequency data. The sampling interval was 1 s.

The procedure used for measurements was as follows. The initial background frequency (signal) was allowed to stabilize for 5 min. Next, a sample of the studied protein was pumped through the cell for 3 min to form the affinity complex. Finally, regeneration of the sensing surface was achieved by flowing buffer through the cell for 2 min (Supplementary Figure S1).

Kinetic parameters were determined using a standard approach (Skladal, 2003) according to following equation:

(2) $$f=f_{0}+f_{\mathrm{eq}}\exp(-k_{\mathrm{obs}}t)$$

The non-linear least squares method was used to obtain values for the parameters *f*_eq_ and *k*_obs_. Furthermore, the kinetic association (*k*_a_) and dissociation (*k*_d_) rate constants were calculated by linear regression assuming a linear dependence of *k*_obs_ on the concentration of cryptogein, *c*_cry_:

(3) $$k_{\mathrm{obs}}=k_{a}c_{\mathrm{cry}}+k_{d}$$

Finally, values of the kinetic equilibrium association (*K*_A_) and dissociation (*K*_D_) constants were calculated from

(4) $$K_{d}=1/K_{A}=k_{d}/k_{a}$$

All calculations were carried out using the curve fitting module provided within the Origin 8.5 software package (Microcal, Northampton, MA, USA).

## Results

### Characterization of Mutants

**FIGURE 1:**
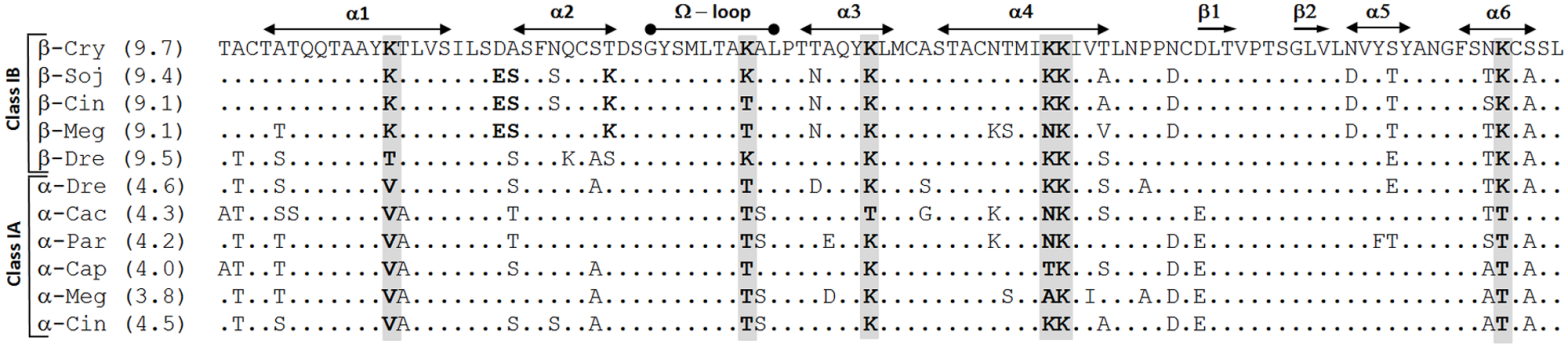
**Comparison of selected α- and β-elicitins.** Multiple sequence alignment of selected protein sequences of elicitins to that of cryptogein was performed using the CLUSTALW algorithm; the corresponding lysine residues are highlighted in bold.

Seven recombinant cryptogeins carrying the following mutations were prepared: Lys39Thr, Lys48Thr, Lys61Thr/Asn70Asp/Asp73Glu (later referred as Lys61Thr) and Lys94Thr, two double mutants Lys39Thr/Lys94Thr and Lys48Thr/Lys94Thr and a triple mutant Lys39Thr/Lys48Thr/Lys94Thr. The exchange for Thr residue was chosen due to the fact that it’s commonly present at these positions in acidic elicitins (**Figure 1**). Moreover, Lys61Thr mutation was introduced with additional mutations Asn70Asp and Asp73Glu commonly present at these positions in acidic elicitins (**Figure 1**) whose role in stabilization of α4-helix could be expected. The MW of the proteins determined by MALDI MS are shown in **Table 1**. The measured values were in good agreement with the calculated ones and confirmed the presence of three disulphide bridges in the structure. The overall structure of the proteins was verified by circular dichroism (CD) spectroscopy, which showed the presence of the typical elicitin fold with ca. 50% α-helix and little β-structure (Gooley et al., 1998). The impact of the mutations on the protein pI was measured by isoelectric focusing (IEF) on IPG readystrips 3–10 (Bio-Rad, USA). The measured values were in good agreement with the theoretical values (**Table 1**). Further, the effect of the mutations on the proteins’ stability was measured by TSA which is a rapid and simple technique for assessing the thermal stability of proteins (Nettleship et al., 2008). As expected from previous observations regarding elicitin stability, cryptogein showed a very high thermal stability, with *T*_m_ of 60.59°C (**Table 1**, Supplementary Figure S2). Mutants Lys39Thr and Lys48Thr showed a slightly higher stability compared to cryptogein, whereas mutant Lys94Thr and Lys61Thr had a considerably lower stability (**Table 1**, Supplementary Figure S2). In the remaining double and triple mutants, no melting curve was measurable. One reason for this could be the difference in protein surface charge affecting binding of SYPRO Orange dye or low stability of proteins because it was also not possible to measure a melting curve for the α-elicitin capsicein as well (Supplementary Figure S2).

**Table 1:** Physical properties of the studied proteins.

Protein	Mass (Da)	pI (Det.)	pI (Calc.)	*T*_m_ (°C)	Rate of sterol transfer (%)
β-Cryptogein	10385	9.8	9.7	60.59 ± 0.03	100
K39T	10357	9.4	9.5	61.46 ± 0.19	67
K48T	10356	9.7	9.5	61.84 ± 0.08	61
K94T	10356	10	9.5	50.20 ± 1.04	135
K39T/K94T	10328	8.7	9.1	ND	65
K61T/K62T	10329	7.1	9.1	ND	59
K39T/K48T/K94T	10301	6.7	6.7	ND	66

*The molecular mass of the proteins was determined by MALDI-TOF analysis. pI values were calculated by EMBOSS explorer (http://emboss.bioinformatics.nl) with three disulfide bridges or were measured by the IEF method on IPG strips (3–10). *T*_*m*_ values, which reflect the thermodynamic stability of the studied protein, were measured by the protein thermal shift assay (TSA) using the SYPRO Orange fluorescent dye. The rate of sterol transfer was measured from the increase in fluorescence after 2 min due to dilution of DHE in the stigmasterol micelles. ND, not detected.*

One of the important physiological features of elicitins secreted by *Phytopthora* species is their extracellular sterol carrier activity, enabling sterols to be taken up from the biological membranes of the host plant (Ponchet et al., 1999). To evaluate the ability of mutants to transfer sterols, the fluorescence increase following addition of elicitins to donor (DHE) and acceptor (stigmasterol) micelles was monitored as described previously (Dokladal et al., 2012). The initial rate of fluorescence increase (Supplementary Figure S3) was evaluated by numerical integration of the time-dependent fluorescence curves and then compared to that of wt cryptogein. Whereas the mutant Lys94Thr exhibited a higher rate (135%) of sterol transfer than wt cryptogein, the other mutants showed lower rates of sterol transfer in the range of 59–67 % (**Table 1**).

### Necrotic Activities of Mutated Cryptogeins

To measure effects of mutations on the induction of necrosis in tobacco plants three widely used methods of protein application were used: (i) direct infiltration into leaves, (ii) application on the stem of decapitated plants, and (iii) application by aspiration through petiole of detached leaves.

Direct infiltration of 5 nM proteins into leaves induced necrosis in approximately 60% of the infiltrated area. Only in the case of mutants Lys61Thr, Lys39Thr/Lys94Thr, and Lys48Thr/Lys94Thr the necrotic area was lower, i.e., 39, 47, and 52%, respectively (**Table 2**). After application of 2.5 μg of proteins on the stem of decapitated plants a similar extent of necrosis in case of wt cryptogein and single mutants Lys61Thr and Lys94Thr was observed. In contrast, reduced induction of necrosis in proteins containing Lys39Thr mutation was observed, but the most pronounced difference was remarked between double mutants carrying mutations in Lys94Thr and Lys39Thr or Lys48Thr (**Table 2**). A similar reduction in necrosis was observed when mutant elicitins were applied via leaf petiole. Mutant Lys39Thr induced only 20% necrosis, mutants Lys48Thr and Lys48Thr/Lys94Thr induced necrotic spots and no induction of necrosis was observed in the double- and triple-mutants carrying mutation Lys39Thr (**Table 2**, Supplementary Figure S4).

**Table 2:** Extent of leaf necrosis.

Protein	Necrosis ± SE (%) Leaf inf. 5 nM (*n* = 4)	Necrosis ± SE (%) Stem app. 2.5 mg (*n* = 4)	Necrosis ± SE (%) Petiole app. 250 ng (*n* = 4)
β-Cryptogein	63.0 ± 7.0	9.8 ± 1.3	29.6 ± 3.5
K39T	60.1 ± 8.4	7.0 ± 1.2	6.2 ± 2.5
K48T	61.1 ± 6.1	8.1 ± 1.4	Spots
K61T/N70D/D72E	39.0 ± 4.7	9.0 ± 2.8	16.3 ± 3.1
K94T	67.9 ± 9.7	9.6 ± 1.3	21.1 ± 2.9
K39T/K94T	46.9 ± 9.2	2.9 ± 1.2	ND
K48T/K94T	52.2 ± 6.1	6.6 ± 1.3	0.7 ± 0.5 (Spots)
K39T/K48T/K94T	58.1 ± 7.2	2.1 ± 0.7	ND

*Three different types of application of the studied proteins were used: direct leaf infiltration, aspiration through petiole of detached leaves and stem application. The extent of leaf necrosis was analyzed by using the ImageJ program. ND, not detected.*

### Accumulation of Defense Gene Transcripts and Resistance to *Phytophthora parasitica*

**FIGURE 2:**
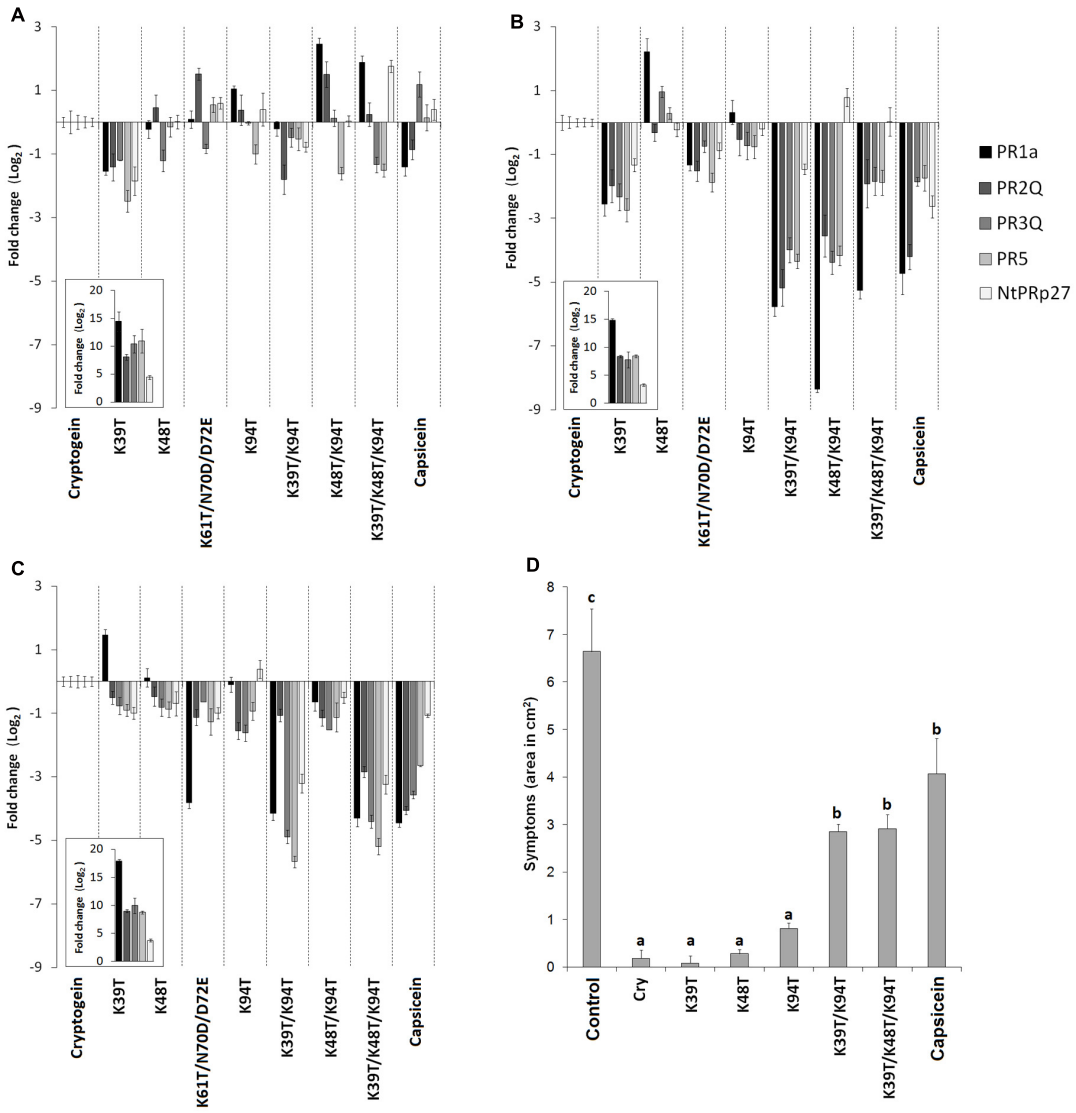
**Accumulation of defense-related genes and induction of resistance against *Phytophthora parasitica* in tobacco plants.** Effect of wt cryptogein and the mutants on the accumulation of transcripts for PR proteins monitored in the directly infiltrated **(A)** and surrounding area **(B)** of leaves or after protein aspiration through petiole of detached leaves **(C)**. Insets represent accumulation of transcripts of wt cryptogein related to control. Gene expression relative to a control was calculated by the ΔΔC(t) method 48 h after proteins application. The values given in the graphs are the log_2_R ratio of the relative increase and its standard deviation (SD). A greater than twofold change in transcript accumulation was taken as significant. **(D)** Eight-week-old tobacco plants were decapitated and treated with elicitins. After 24 h non-necrotic parts of leaves were inoculated with zoospores of *P. parasitica*. The invaded areas were measured 3 days after inoculation. Each bar represents the standard error of four replicates from three different experiments. Each replicate corresponded to eight inoculated areas on four leaves from one plant. Student’s *t*-test with *p* = 0.01 was used to determine whether differences in the area were statistically significant. Different letters denote a significant difference. Cry = wt cryptogein.

The relationship between the performed mutations and transcript levels for selected PR proteins was evaluated by RT-qPCR assays. Based on the results of a previous study (Dokladal et al., 2012), the following pathogenesis related genes were selected for transcript quantification: *PR1a, PR2Q, PR3Q, PR5*, and *NtPRp27 (PR17)*. Transcript levels of all these genes were related to those induced in a wt cryptogein sample by the ΔΔCt method. Individual transcripts were quantified in leaves directly infiltrated with proteins or after protein aspiration through petiole of detached leaves. In case of leaves directly infiltrated with proteins, transcript levels were determined in both, the infiltrated and the surrounding area which was 5 mm in width. For mutated cryptogeins at the site of infiltration, only a slight change in transcript levels was observed compared to those observed for cryptogein (**Figure 2A**). In contrast to double mutant Lys48Thr/Lys94Thr, the double mutant Lys39Thr/Lys94Thr and the triple mutant Lys39Thr/Lys48Thr/Lys94Thr showed a considerable decrease in transcript levels in the surrounding area and after aspiration through petiole of detached leaves (**Figures 2B,C**).

The results of the transcript analysis presented above suggest that the introduction of some mutations to cryptogein may reduce the level of resistance induced. To verify this, the resistance of tobacco plants to oomycete *P. parasitica* was evaluated for selected mutant variants. Eight-week-old tobacco plants were decapitated and treated with elicitins and after 24 h leaves were inoculated with zoospores of *P. parasitica.* As expected, there was significant reduction in induction of resistance for plants treated with the Lys39Thr/Lys94Thr double mutant and triple mutant Lys39Thr/Lys48Thr/Lys94Thr comparable to that of typical acidic elicitin capsicein (**Figure 2D**).

All these results are in a pretty relation to results from necrotic activities of proteins after application on the stem of decapitated plants or aspiration through petiole of detached leaves and sustain a generally accepted hypothesis that systemic resistance induced by elicitins derives from their presence mediated by systemic movement across the plants (Devergne et al., 1992; Keller et al., 1996). Moreover, in this process the role of some specific lysine residues seems to be more pronounced. On the base of these findings we decided to study in more detail the effect of mutations on the movement of proteins within the plants.

### The Effect of Mutations on Movement of Cryptogein Through Plants

**FIGURE 3:**
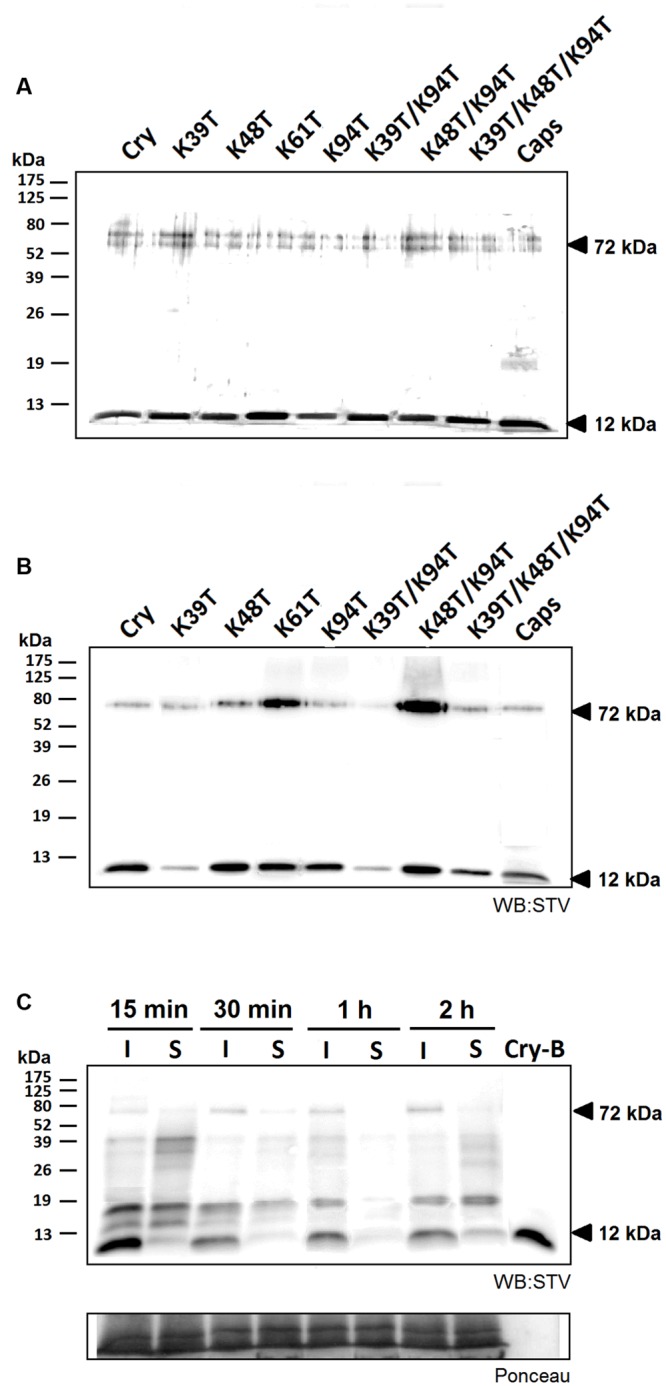
**Efficiency of biotin labeling and its effect on protein movement.** The efficiency of labeling procedure was analyzed by protein detection by silver staining **(A)** and by western blot analysis using streptavidin labeled with horseradish peroxidase and luminescence detection (STV; **B)**. In both cases 500 ng of proteins samples were separated and a small but visible shift in protein migration was detected as well as a significantly compromised labeling efficiency in case of proteins carrying the Lys39 mutation. Furthermore, a 72 kDa band corresponding to formed homohexamer was apparent in all tested proteins. **(C)** Observation of localization of biotin-labeled cryptogein in time. Samples from the site of infiltration and the surrounding tissue from three different leaves were extracted with a phosphate buffer containing 1% SDS and submitted to 15% SDS-polyacrylamide gel electrophoresis followed by western-blot analysis using streptavidin labeled with horseradish peroxidase and chemiluminescence detection (STV). In the first 30 min after infiltration a decrease in the protein concentration is visible, moreover after 2 h a small amount of protein emerged in the surrounding area. Control (CTRL) represents the samples without any treatment and Cry-B represents 5 ng of biotin-labeled wt cryptogein.

**FIGURE 4:**
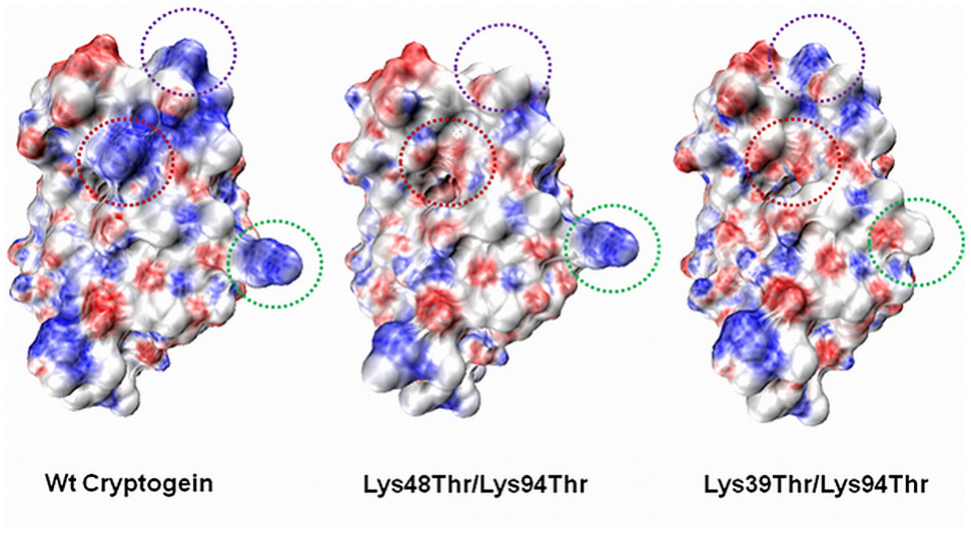
**Electrostatic potential surface maps of wt cryptogein and double mutants Lys48Thr/Lys94Thr and Lys39Thr/Lys94Thr.** The Lys39 residue in the omega loop region is one of the most exposed lysine residue and Lys39Thr mutation significantly alters the distribution of charges within the omega-loop of cryptogein. The positions of the mutated residues are indicated by the green circle (Lys39), purple circle (Lys48), and red circle (Lys94). The surfaces were calculated and displayed using PyMol v0.99 (DeLano Scientific).

To observe the movement of studied proteins in plants, cryptogein mutants and a typical acidic elicitin capsicein were labeled with biotin. The efficiency of labeling procedure was analyzed by comparison of results from protein detection by silver staining and by western blot analysis using streptavidin labeled with horseradish peroxidase and luminescence detection. The results showed a little shift in migrations of biotinylated proteins and significantly compromised labeling efficiency in mutants lacking the Lys39 residue (**Figures 3A,B**). This phenomenon could be explained by the fact that Lys39 residue in omega-loop region is one of the most accessible lysine residues for biotin-labeling and thus its absence reduces labeling efficiency (**Figure 4**). In addition, a 72 kDa band corresponding to homohexamer was apparent in all tested proteins (**Figures 3A,B**, respectively) with apparently higher labeling efficiency in mutants Lys48Thr, Lys48Thr/Lys94Thr, and Lys61Thr. This finding is not so surprising because recently the heterohexamer formation of POD1/POD2 proteins from *Pythium oligandrum* exhibiting similar structure to that of elicitins was proved (Takenaka et al., 2011). However, in contrast to POD1/POD2D heterohexamer structure the covalent linking of monomers in detected cryptogein homohexamer structure should be assumed regarding the fact of proteins analysis by denaturing SDS-PAGE under reducing conditions.

Biotinylated proteins were introduced to plants by aspiration through petiole of detached leaves (1 μg of protein) or by direct infiltration into leaf mesophyll using a syringe (50 nM solution). In case of direct infiltration we first monitored the localization of wt cryptogein 15 min, 30 min, 1 h, and 2 h after application (**Figure 3C**). Total proteins were extracted to phosphate buffer with 1% SDS from infiltrated tissue and surrounding tissue which was 3 mm in width and submitted to SDS-polyacrylamide gel electrophoresis followed by western-blot analysis using streptavidin labeled with horseradish peroxidase and chemiluminescence detection. A decrease in cryptogein concentration was visible within the first 30 min after infiltration and after 2 h a small amount of protein appeared in the surrounding area. Such decrease could be explained by insolubilization of the cryptogein in the cell wall resulting in a loss of its extractability in used buffer as shown previously (Dorey et al., 1997).

**FIGURE 5:**
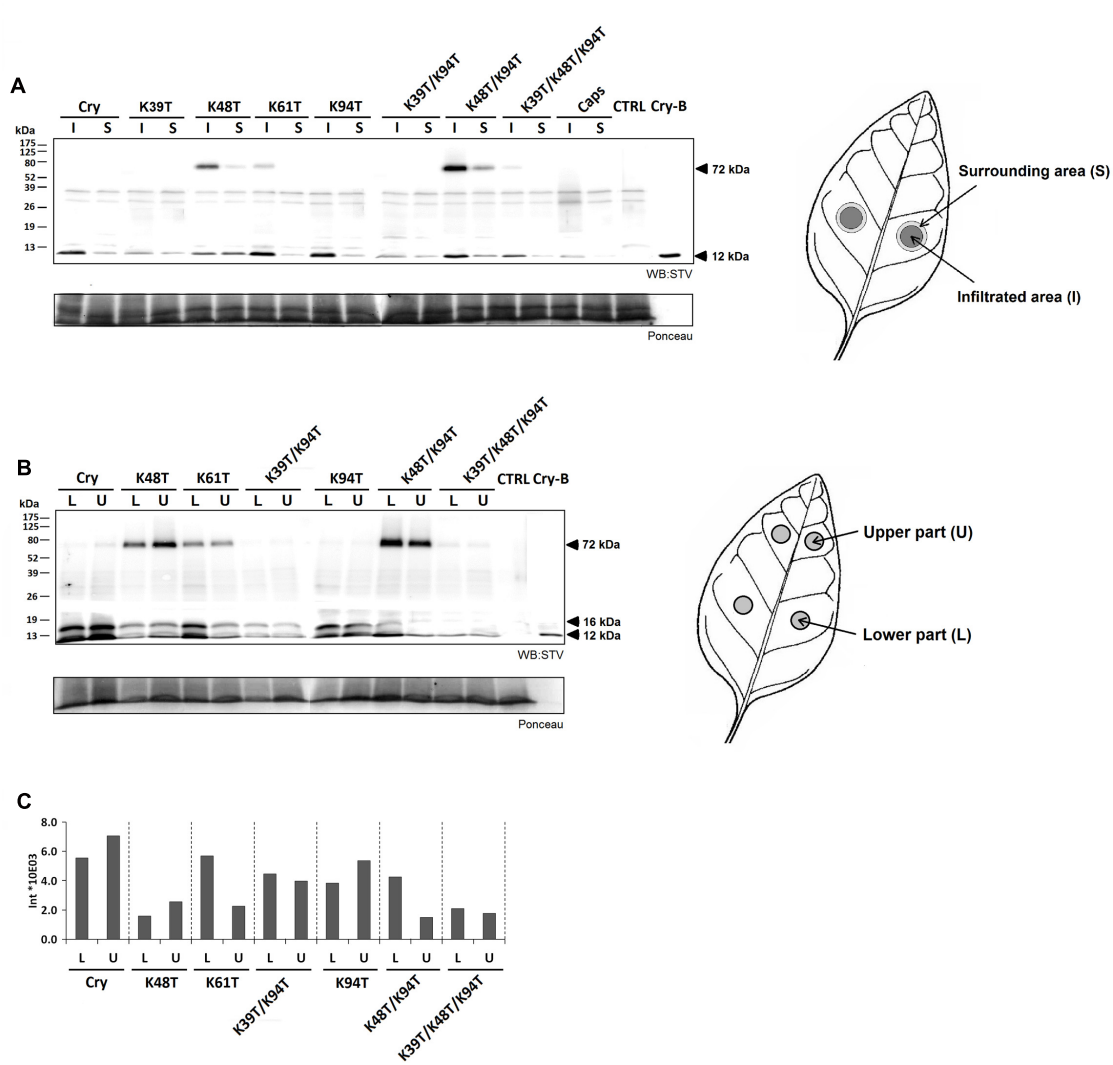
**Localization of labeled cryptogein after direct infiltration into tobacco leaves and after aspiration though leaf petiole. (A)** Three hours after direct infiltration of biotin-labeled capsicein (Caps), cryptogein (Cry) and its variants into leaf mesophyll of three different leaves, samples from the site of infiltration and the surrounding area were collected, extracted with phosphate buffer with 1% SDS and submitted to 15% SDS-polyacrylamide gel electrophoresis followed by western-blot analysis using streptavidin labeled with horseradish peroxidase and chemiluminescence detection (STV). Except for the mutant K48T all proteins showed higher concentration at the zone of infiltration in comparison with the surrounding zone. **(B)** Six hours after aspirating of biotin-labeled cryptogein (Cry) and its variants through petiole of detached leaves to three different leaves, samples from two different regions corresponding to the lower and upper part of the leaf were collected. Majority of the proteins exhibited approximately equal distribution in both, lower and upper parts of the leaf, whereas K61T and K48T/K94T mutants showed a little higher concentration in the lower part than in the upper part of the leaf. In addition, a second band with molecular mass of about 16 kDa was detected in all proteins suggesting a possible modification of proteins in plants. **(C)** Intensities of biotin-labeled cryptogein and its variants 6 h after aspirating through leaf petiole of detached leaves recalculated according to different efficiency of biotinylation reaction (**Figure 3**). Control (CTRL) represents the samples without any treatment and Cry-B represents 5 ng of biotin-labeled wt cryptogein.

Using this assay the localization of other studied cryptogein mutants was monitored 3 h after infiltration to leaves. Mutants lacking residue Lys39 are less visible due to the low labeling efficiency mentioned above. Faint signals about 32 and 37 kDa, detected in all samples including control, could correspond to known endogenous biotinylated proteins such as biotin carboxyl carrier protein with molecular mass about 35 kDa. With an exception of K48T mutant, all the proteins were present at higher levels in the zone of infiltration compared to the surrounding zone. In case of triple mutant and capsicein, only a very faint or no band was detected (**Figure 5A**) in surrounding zone. This result could be consistent with the work of Dorey et al. (1997) who showed a strict localization of acidic ^125^I labeled H20 glycoprotein from *P. megasperma*, belonging to class III of elicitins, to the infiltrated zone. In addition, it was observed that infiltration of basic elicitins, but not acidic ones, in high concentration often caused necrosis beyond the site of infiltration.

In case of application by aspiration through petiole, the distribution of selected biotin-labeled proteins was monitored in two different regions corresponding to the lower and upper part of the leaf, respectively (**Figure 5B**). After recalculation of results to different biotinylation efficiency, in both analyzed regions a significantly lower level of proteins was observed in mutants containing Lys48Thr mutation (**Figure 5C**). In case of mutants Lys48Thr and Lys48Thr/Lys94Thr this could be related to high amount of detected homohexamer (**Figure 5B**) and explains low extent of necrosis demonstrated as necrotic spots (**Table 2**). Distribution of proteins in both, lower and upper parts of the leaf, was approximately equal when only Lys61Thr and Lys48Thr/Lys94Thr mutants exhibited lower concentration in the upper part of the leaf corresponding to observed necrosis mainly in the lower part of the leaves (**Figure 5C**, Supplementary Figure S4). Noticeably, in all proteins a second band with molecular mass of about 16 kDa (identified by LC-MS/MS as cryptogein) was detected. This result suggests some modification of proteins in plants when more detailed characterization of this potential modification is on-going in our laboratory. The above results indicate that movement of proteins from application zone to distant zone is not considerably affected by the protein charge, as shown previously for the radioiodinated α- and β- elicitins within tobacco plants (Devergne et al., 1992) but the role of some lysine residues is obvious. Recently, it has been well demonstrated that properties of exogenous proteins could affect their ability to enter and exit phloem (Niu et al., 2011). Hence, the presence of specific lysine residues could be important for the ability of elicitins to unload phloem via symplastic or apoplastic pathway.

## The Role of Homodimer

**FIGURE 6:**
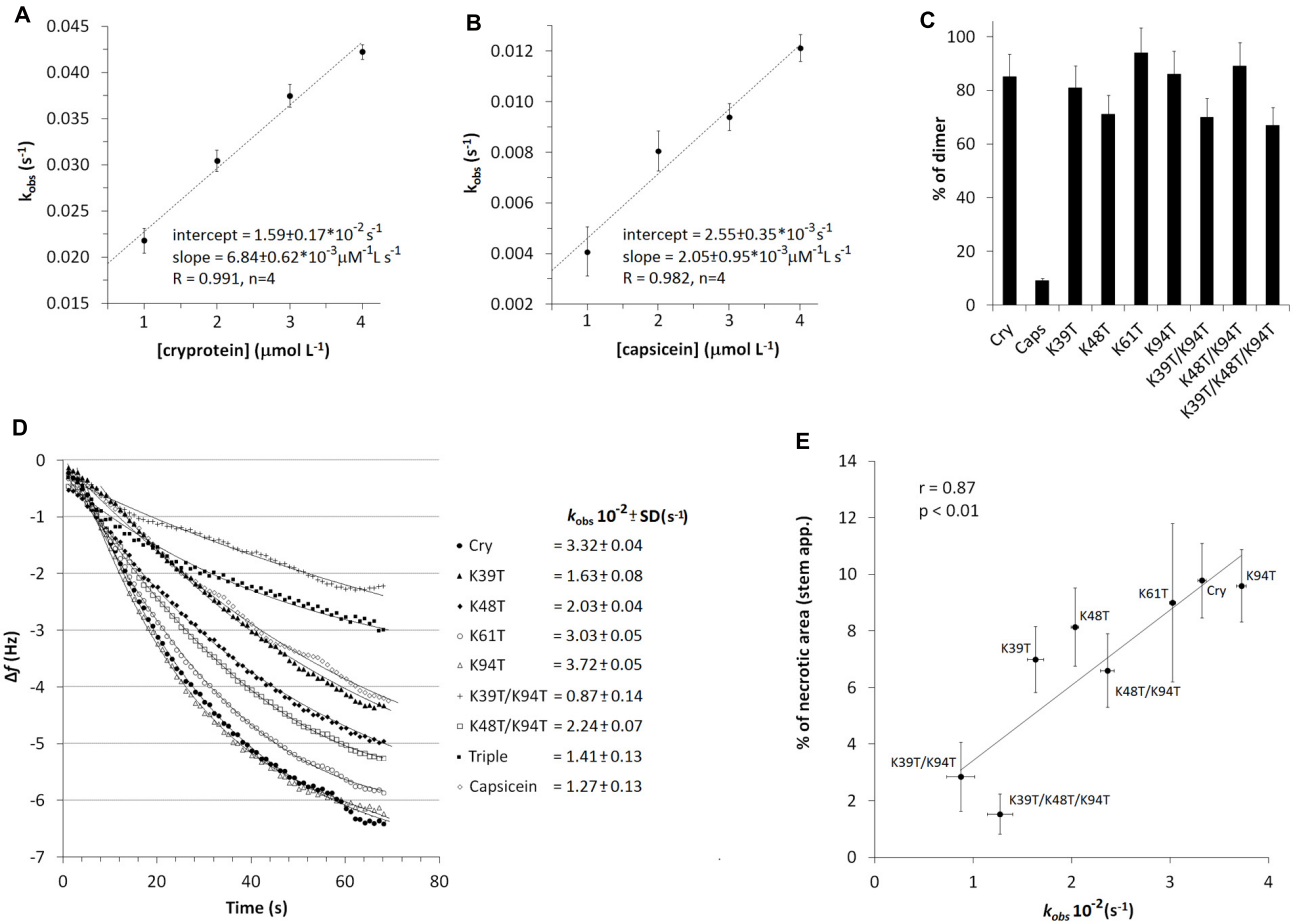
**Effects of mutation on dimer formation.** Plotting of calculated *k*_obs_ values (Eq. 2) against the molar concentration of proteins enabled a linear fit to the data (*n* = 3), where the slope and intercept directly corresponded to the kinetic rate constants for association (*k*_a_) and dissociation (*k*_d_) of cryptogein **(A)** and **(B)** capsicein, representing a typical β- and α-elicitin, respectively (Eq. 3). **(C)** Evaluation of dimer/monomer rate in percentage formed after EGS mediated dimer crosslinking. Individual proteins were crosslinked with EGS reagent, separated by 15% SDS-PAGE and then the amount of monomer and dimer was calculated from band intensities after silver staining. **(D)** Comparison of binding rates (*k*_obs_) of cryptogein (and its) variants forming (homo- and hetero-) dimers at 2 μM concertation. **(E)** Correlation of obtained kinetic constants of dimer formation with induced necrosis after stem application.

**FIGURE 7:**
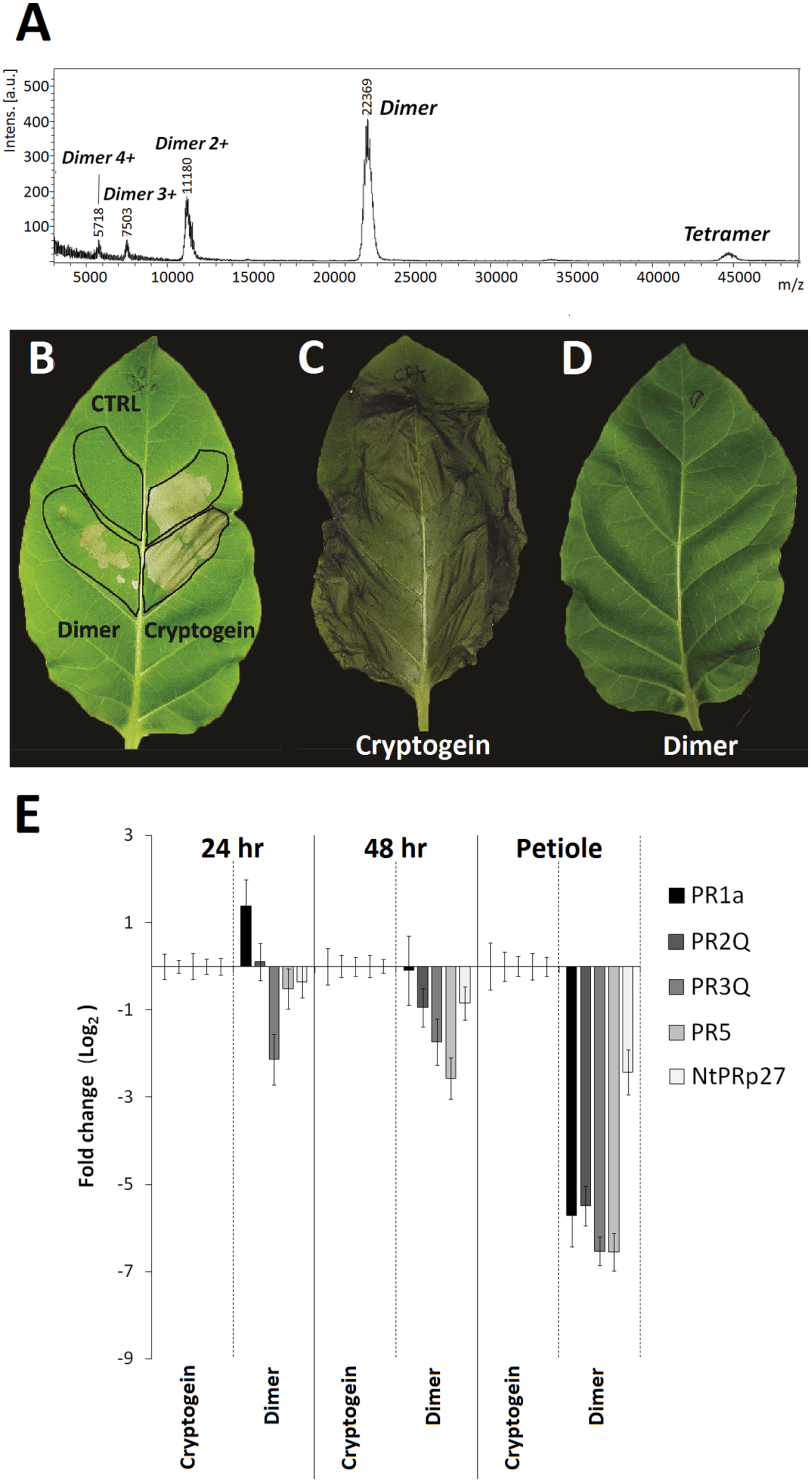
**Biological activity of cryptogein dimer on tobacco plants.** MALDI-MS spectrum shows crosslinked dimer and a low amount of potentially crosslinked tetramer **(A)**. Necrotic activity of cryptogein and its crosslinked dimer after direct infiltration **(B)** and aspiration through petiole of detached leaves **(C,D)**. Effect of wt cryptogein and its crosslinked dimer on the accumulation of transcripts for PR proteins monitored in the directly infiltrated area of leaves or after protein aspiration through petiole of detached leaves **(E)**. Gene expression relative to a control was calculated by the ΔΔC(t) method 24 and /or 48 h after proteins application. The values given in the graphs are the log_2_R ratio of the relative increase and its standard deviation (SD). A greater than twofold change in transcript accumulation was taken as significant.

One of the important parameters in this process could be a dimerization of elicitins which has been suggested to play a role in their biological activity (Ponchet et al., 1999) and it has been shown to occur on the same side of the protein as the functionally important residue Lys13 (Pleskova et al., 2011). To evaluate the monomer/homodimer rate in solution and to determine the homodimer biological activity a crosslinking experiment was done. Based on the published X-ray structure of cinnamomin homodimer (Rodrigues et al., 2006), showing a distance between Lys13 of one monomer and Lys62 of the other monomer about 18 angstroms (Supplementary Figure S5), the EGS (ethylene glycol bis[succinimidylsuccinate]) crosslinking reagent was used. The results from crosslinking experiment showed that the pre-dominant form in solution of all tested proteins was a homodimer while capsicein lacking the residue Lys13 was not crosslinked at all (**Figure 6C**). To measure biological activity of wt cryptogein homodimer, the crosslinked protein was purified by HPLC on reverse phase (**Figure 7A**) and its activity was tested after direct infiltration into leaves (10 nM solution of protein) and after aspiration through petiole of detached leaves (1 μg of protein). Even though direct infiltration of homodimer induced only faint necrotic symptoms, levels of selected transcripts were comparable with those induced by wt cryptogein (**Figure 7B**). On the other hand, application of homodimer by the aspiration through petiole of detached leaves induced no necrotic symptoms and compared to wt cryptogein (**Figures 7C,D**), a considerable decrease in transcript levels resembling to that of capsicein was observed (**Figure 7E**). All these data sustained the important role of homodimer in elicitins’ biological activity so we decided to characterize the kinetics of homodimer formation in more detail.

### The Role of Mutations in Dimerization Kinetics

To monitor the process of dimer formation piezoelectric sensor was employed because it has been shown to be a convenient tool for real-time bio-interaction studies (Skladal, 2003). Piezoelectric effect is formed by mechanical action on certain anisotropic crystals (e.g., quartz), wherein oriented dipoles are formed. The phenomenon works also in reverse, after applying a suitable alternating voltage frequency crystal starts to vibrate. Binding of the test compound to the crystal surface reduces the resonant frequency of the crystal and vice versa. The drop in frequency is proportional to the mass of bound substance.

First, cryptogein and capsicein representing typical β- and α-elicitins, respectively, were covalently attached to the crystal surface via its amino groups (lysine residues). The association of proteins to the piezoelectric sensor resulted in a decrease in sensor’s frequency due to the formation of surface-bound affinity complexes (Supplementary Figure S1 shows a typical trace obtained in the dimerization experiments). To study the kinetics of dimer formation, a series of solutions with different concentrations of cryptogein and capsicein were used. Fitting of the measured decrease in frequency versus time to Eq. 2 using non-linear regression yielded the value of *k*_obs_ for each concentration of proteins used. Plotting of these *k*_obs_ values against the molar concentration of proteins enabled a linear fit to the data (**Figures 6A,B**), where the slope and intercept directly corresponded to the kinetic rate constants for association (*k*_a_) and dissociation (*k*_d_) (Eq. 3). The values of the kinetic rate constants and corresponding kinetic equilibrium constants for dimer formation are shown in **Table 3**. The measured values of rate and equilibrium constants for both elicitins are much closer to physiological conditions than calculated previously (Gooley et al., 1998) showing a little higher strength of the interaction in capsicein homodimer hardly explaining so different behavior.

**Table 3:** Kinetic parameters of elicitin dimer and nsLTP1-elicitin complex formation.

Interacting proteins	*k*_a_	*k*_d_	*K*_a_	*K*_d_
	(M^-1^ s^-1^)	(s^-1^)	(M^-1^)	(M)
Cry-Cry	6.99 ⋅ 10^-3^	1.54 ⋅ 10^-2^	4.53 ⋅ 10^5^	2.21 ⋅ 10^-6^
Caps-Caps	2.23 ⋅ 10^-3^	3.12 ⋅ 10^-3^	7.15 ⋅ 10^5^	1.40 ⋅ 10^-6^

*The kinetic association, dissociation and equilibrium constants for the selected interactions were calculated from the values of slope and intercept indicated in **Figures 6A,B**.*

Consequently, we decided to test the effect of individual lysine mutations on ability of proteins to form dimer structure with wt cryptogein covalently attached to the crystal surface via its amino groups. Solutions with different cryptogein variants at 2 μM concentration were used and to allow comparison of the binding rates to the *k*_obs_ constants obtained previously (**Figure 6D**). The same density was used on the sensing surface as used for wt cryptogein. Only the mutant Lys94Thr showed a comparable rate of dimer formation to that of wt cryptogein. In all other mutants, a significantly lower rate of dimer formation was measured. Most strikingly, the plotting of *k*_obs_ values representing binding rates correlated very well with the necrosis after stem application with a noticeable role of the residue Lys39 in double mutant Lys39Thr/Lys94Thr and triple mutant Lys39Thr/Lys48Thr/Lys94Thr (**Figure 6E**).

Founded results indicate that the ability of elicitins to induce distal resistance could be orchestrated by another partner in plants whose interaction takes place on the same side of the protein as the interaction between monomers. In plants one of the best potential candidates are lipid transfer proteins (LTPs) exhibiting similar properties to elicitins such as having mainly basic pI and assumed cell wall localization (Carvalho and Gomes, 2007). In addition some helices of nsLTP1s and elicitins in three-dimensional space were superimposed (Blein et al., 2002) and competition of nsLTP1 and elicitins for the same membrane receptor was proved. They are likely to play an important role in key processes of plant physiology when recently their involvement in plant defense signaling has emerged (Champigny et al., 2013).

### Interaction of Studied Proteins with Lipid Transfer Proteins

**FIGURE 8:**
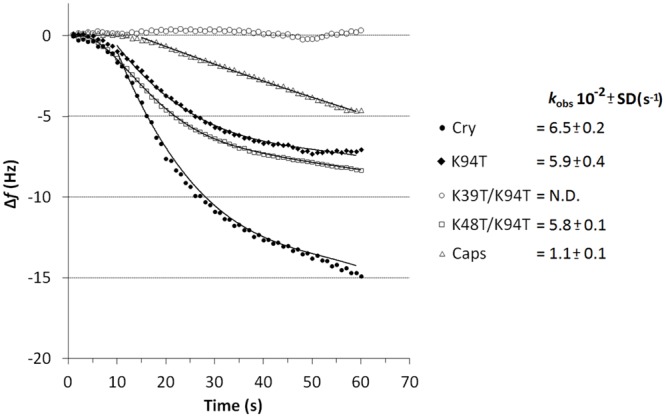
**Evaluation of nsLTP1-elicitin complex formation curves measured by piezoelectric biosensor QCM.** Comparison of affinities of immobilized nsLTP1 for capsicein, wt cryptogein and its biologically active (K94T, K48T/K94T) and low active (K39T/K94T) mutants in solution (protein concentration of 10 μM). The apparent binding constants *k*_obs_ (*n* = 3) describe the rate of formation of the cryptogein-nsLTP1 complex.

To monitor potential interaction of LTPs with cryptogein piezoelectric sensor was employed as well as in case of elicitin dimer formation measurement when previously well characterized nsLTP1 from wheat (Blein et al., 2002) was covalently attached to the crystal surface. To clear-up the role of lysine residues in LTP-elicitin interaction measurement with wt cryptogein, biologically active mutants Lys94Thr and Lys48Thr/Lys94Thr and low active mutant Lys39Thr/Lys94Thr and capsicein representing typical acidic elicitin was done. Fitting of the measured decrease in frequency versus time to Eq. 2 using non-linear regression yielded the value of *k*_obs_ for each protein used (**Figure 8**). In contrast with double mutant Lys39Thr/Lys94Thr all other proteins interacted with nsLTP1 (**Figure 8**). Cryptogein variants Lys94Thr and Lys48Thr/Lys94Thr provided significantly, but only slightly, lower rate of complex formation but in case of capsicein the binding rate was six times slower compared to the wild-type cryptogein. Noticeable is the appealing connection between the measured rate of nsLTP1-elicitin complex formation and proteins’ biological activity in term of necrosis and resistance induction after the stem application.

## Discussion

### The Role of Mutations on Proteins’ Stability

Recent studies have indicated that elicitin activity depends on the presence of specific lysine residues, as demonstrated by the observed correlation between necrotic index and pI (Pernollet et al., 1993; Pleskova et al., 2011; Dokladal et al., 2012). We carried out site-directed mutagenesis on β-elicitin cryptogein to replace five selected lysine residues with threonine residues, which often occur in place of lysine residues in the elicitin primary structure (**Figure 1**). Interesting results were obtained regarding the thermal stability of the measured proteins because it was not possible to obtain a melting curve for all double mutants and the triple mutant. One explanation for this could be low stability of the proteins. However, this was contradicted by further measured results. Another explanation could be disruption of SYPRO orange dye binding due to the change in protein charge. This assumption seems to be more probable, especially as it was also difficult to measure a melting curve for the natural acidic elicitin capsicein secreted by oomycete *P. capsici*.

### Sterol Transfer Activity of Mutants

Even though an important role of conformation change in the ω-loop for elicitin activity driven by sterol binding has been suggested (Osman et al., 2001), our results support recent findings that sterol binding is not crucial for determining elicitin activity (Dokladal et al., 2012). In general, the sterol transfer activity of the mutants did not correlate with their biological activity and measured lower capacity of mutants to transfer sterol is probably influenced by changes in the protein surface charge, which can disrupt the correct positioning of the protein during the process of sterol transfer from biological membranes, as discussed previously (Pleskova et al., 2011). One interesting exception was the mutant Lys94Thr, which showed a higher rate of sterol transfer, mainly due to better exchange kinetics.

### Necrotic Activity of Mutants

Determination of the necrotic activity of the mutated proteins demonstrated a fact that biological activity of elicitins strongly depends on application method. No correlation was detected between the data obtained after application of mutated proteins onto decapitated stem or aspiration through petiole of detached leaves, or by direct infiltration to the leaf apoplast. A positive correlation between the pI of the mutated proteins and necrosis induced after their application onto decapitated stem or aspiration through petiole of detached leaves supports a previous suggestion that mutations of lysine residues to neutral amino acids may result in lower toxicity of β-elicitins in terms of necrosis (Ponchet et al., 1999). Moreover, a correspondence of necrotic symptoms on leaves after stem/petiole application with resistance against *P. parasitica* and transcript levels of selected PR genes sustain the previous suggestion that necrosis is not required for successful protection but strongly enhances the resulting protection level (Ponchet et al., 1999). However, noticeable differences in necrotic activity after mutation of individual lysine residues (especially in the case of aspiration through petiole of detached leaves) suggest that this interpretation should be treated with caution. In particular, lysine residue 13 seems to play a special role as mutation to Val has been shown to significantly decrease protection against *P. parasitica* on tobacco plants (Pleskova et al., 2011), as well as lead to reduced ability to induce necrosis after application onto decapitated stem (Pleskova et al., unpublished results). In addition, the role of other residues, such as Lys 39, is obvious, especially in case of double mutants carrying the Lys94Thr mutation. Interestingly, in all basic elicitins, mutation of Lys 39 to a Thr residue is connected with mutation of residue Thr 29 for Lys (**Figure 1**).

### The Role of Proteins Movement within the Plant

Interesting results were obtained regarding the analysis of transcripts in the area surrounding the site of infiltration responsible for LAR. The results were almost the same as those obtained after aspiration through petiole of detached leaves, with a marked decrease of transcripts in double mutant Lys39Thr/Lys94Thr, the triple mutant and capsicein. Previously, a strict localization of elicitin H20 glycoprotein to the infiltrated zone has been shown, but the endogenous signaling mechanism responsible for triggering the defense responses in cells surrounding the elicitor-treated tissue has yet to be resolved (Dorey et al., 1997). On the other hand, infiltration of elicitin cryptogein, but not acidic elicitin capsicein, in high concentration often causes necrosis beyond the site of infiltration. Owing to the close connection between necrosis and elicitin presence, partial diffusion of elicitin cryptogein outside the infiltration site is likely to occur. Results from localization of biotinylated protein after direct infiltration into leaf support this hypothesis when almost non-detectable concentration of acidic elicitin capsicein in the surrounding tissue could correspond to previous results from acidic H20 glycoprotein.

No clear relation between the distribution of studied mutant proteins across the leaf after aspiration through petiole of detached leaves and their charge demonstrates that the pI of elicitins does not play a crucial role in their movement through the plants by sieve elements. This finding is consistent with one of the first publication studying the migration of radioiodinated α- and β- elicitins within tobacco plants (Devergne et al., 1992). However, the other important factors could be the efficiency and pathway of consequent transport from sieve element to companion and parenchyma cells because it has been demonstrated that protein’s properties play an important role in phloem loading/unloading (Niu et al., 2011). This phenomenon would be related to the dependence of biological activity of elicitins on application method. As well, elicitin penetration through the negatively charged cell wall could contribute to delay response of suspension cells to acidic elicitins despite their similar binding parameters to high affinity binding site on the plasma membrane (Bourque et al., 1998).

### The Role of Interaction Partner in Proteins Activity

A clear role of specific lysine residues in the process of elicitin movement and perception within the plant suggests a possible interaction with another partner. Noticeable is the role of Lys39 residue responsible for an absolutely different biological activity of double mutants Lys39Thr/Lys94Thr and Lys48Thr/Lys94Thr. This residue is localized in omega-loop region of the protein near to residue Leu41 for which an important role in the binding process to the potential partner in plant responsible for defense response was proved (**Figure 4**; Dokladal et al., 2012). Moreover, in double mutant Lys39Thr/Lys94Thr the localcharge on one site of protein is significantly affected (**Figure 4**) which could influence possible interactions with other proteins.

One of the possible partners could be the elicitin itself because the ability of elicitins to form dimer under normal conditions was predicted and moreover β-elicitin cinnamomin crystallized as a homodimer (Rodrigues et al., 2006). The results from the quartz crystal microbalance (QCM) and crosslinking experiments confirmed cryptogein homodimer formation in solution with a *K*_D_ value much closer to physiological conditions than calculated previously (Gooley et al., 1998). Surprisingly, covalent crosslinking of cryptogein dimer resulted in its largely reduced ability to induce necrosis and transcript accumulation after aspiration through petiole of detached leaves. However, the measured kinetic parameters of cryptogein dimer formation resembled those of capsicein thus homodimer formation does not seem to be a key process. More interesting results brought the measurement of heterodimer formation kinetics between wt cryptogein and individual cryptogein variants demonstrating a very strong correlation with necrotic activity of proteins after the stem application. Because dimer structure seems to be a major form of proteins in solution this measurement shows proteins capacity to form heterodimer complex instead of homodimer complex and suggests possible interaction of elicitins with other partner in plant. From this point of view the measured interaction of studied proteins with nsLTP1, behaving as an elicitin antagonist with known superimposition of helix H_3_ with H_A_ of cryptogein, is appealing and promotes proposed dialog between LTPs and elicitins in defense signaling (Blein et al., 2002). Moreover, a strong correlation of complex formation kinetic with proteins’ biological activity after stem/petiole application supports rationality of this interaction for future studies. To test this hypothesis, the interaction of tobacco nsLTP1 with wt cryptogein was preliminary tested (for details see supplementary data, Supplementary Figure S6) and detailed characterization of elicitins interaction with nsLTP1 in tobacco plant is ongoing in our laboratory.

Taken together, our work shows that the ability of elicitins to induce resistance in distal tissue is driven by differences in their movement and targeting within the plant and stresses the importance of processes preceding direct elicitin recognition by the plant cells. However, it is obvious that the biological activity of elicitins in term of systemic resistance induction cannot be explained by one specific factor but probably results from combination of several factors. One of the important factors could be elicitins’ capacity to interact with other potential endogenous plants partners, as suspected here for nsLTP1, with a clear involvement of specific lysine residues and corresponding local proteins charge. In this process the residues Lys13 and Lys39 seems to be the most important ones. The other important factor could be the overall surface charge restricting diffusion of the more acidic elicitins at physiological pH in the negatively charged cell wall without altered perception by the cells as suggested previously (Bourque et al., 1998). Nevertheless, in naturally occurring elicitins the both factors are always connected together.

## Author Contributions

JL and TK conceived and designed the experiments with the help of PS. ZZ and OS performed MS experiments and contributed to the preparation of proteins. MO performed the preparation of proteins and analysis of TSA and sterol-transfer assay data. HU with the help of JL and PS analyses raw data from interaction assays. HU and JK performed qRT-PCR and the part on resistance experiments. JL, HU, MO, and TK wrote the manuscript; all authors contributed to the discussion and approved the final manuscript.

## Conflict of Interest Statement

The authors declare that the research was conducted in the absence of any commercial or financial relationships that could be construed as a potential conflict of interest.
