# Supplementary material for: Elicitin-Induced Distal Systemic Resistance in Plants is Mediated Through the Protein–Protein Interactions Influenced by Selected Lysine Residues
Source: Front Plant Sci. 2016 Feb 5;7:59. doi: 10.3389/fpls.2016.00059 (PMC4742723; doi:10.3389/fpls.2016.00059)
Supplement: Supplementary file 1 [file Data_Sheet_1.DOCX]

**Supplementary Information**

**Hana Uhlíková^1^, Michal Obořil^1^, Jitka Klempová^1^, Ondrej Šedo^2,3^, Zbyněk Zdráhal^2,3^, Tomáš Kašparovský^1^, Petr Skládal^1^ and Jan Lochman^1^**

Author Affiliation:

^1^Department of Biochemistry, Faculty of Science, Masaryk University, Kotlářská 2, 61137 Brno, Czech Republic

^2^Research Group Proteomics, Central European Institute of Technology, Masaryk University, Brno, Czech Republic

^3^National Centre for Biomolecular Research, Faculty of Science, Masaryk University, Brno, Czech Republic

**Table S1.** Sequences of the oligonucleotides used for mutagenesis of cryptogein and qPCR primer sequences.

| *GeLiP* | F*: GTCCAAGATTTCTGCGTCGC  R*: tttccagctgcactaagccc |
| --- | --- |
| *NtPRp27* | F*: ATTGTACCACGAGAGCACCCA  R*: GGTTTCACCCAGTGGCTAGGT |
| *PR1a* | F*: CCTCGTACATTCTCATGGTCAAT  R*: CCATTGTTACACTGAACCCTAGC |
| *PR2q* | F*: TCCAGCAGATGTTGTGTCGCT  R*: GGCTTGGCTAGCAGCAACATT |
| *PR3q* | F*: TCTGGATCACCAATGGCATT  R*: AGAAGCCATTGGCAGGACAT |
| *PR5* | F*: CCGAGGTAATTGTGAGACTGGAG  R*: CCTGATTGGGTTGATTAAGTGCA |
| *EF-1α* | F*: TGTGATGTTTTTGTTCGGTCTTTAA  R*: TCAAAAGAAAATGCAGACAGACTCA |
| *K39T* | F*: CCATGCTGACGGCCACGGCCCTCC  R*: GGAGGGCCGTGGCCGTCAGCATGG |
| *K48T* | F*: CCACGGCGCAGTACACGCTCATGTGCG  R*: CGCACATGAGCGTGTACTGCGCCGTGG |
| *K94T* | F*: CGGCTTCTCGAACACGTGCTCGTCGCTGT  R*: ACAGCGACGAGCACGTGTTCGAGAAGCCG |
| *K61T* | F*: CATGCAACACCATGATCACGACGATCGTGA  R*: GTACGTTGTGGTACTAGTGCTGCTAGCACT |
| *N70D/D72E* | F*: TGAACCCGCCCGACTGCGAACTGACGGTGCC  R*: GGCACCGTCAGTTCGCAGTCGGGCGGGTTCA |

* F is the forward primer and R is the reverse primer.


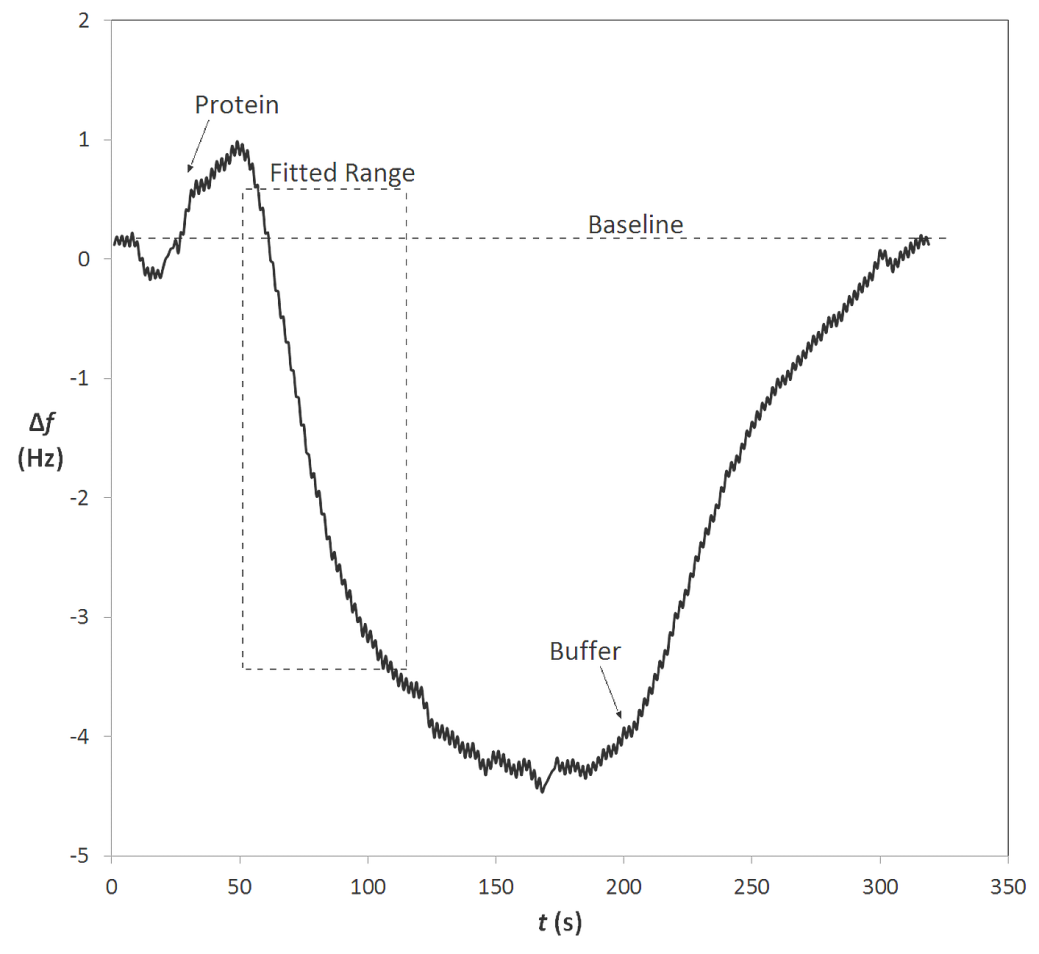


**Figure S1.** *Typical trace of frequency change vs. time obtained in the dimerization experiment.* A piezoelectric crystal with covalently attached cryptogein was initially allowed to stabilize while recording the baseline signal. Next, the studied protein in measuring buffer was introduced. After 30 s, the signal started to increase and the association data were collected for 1 min (indicated by the rectangular area). The protein was allowed to react for 2 min, after which buffer was flowed through the cell to dissociate the dimer and recover the initial baseline signal.


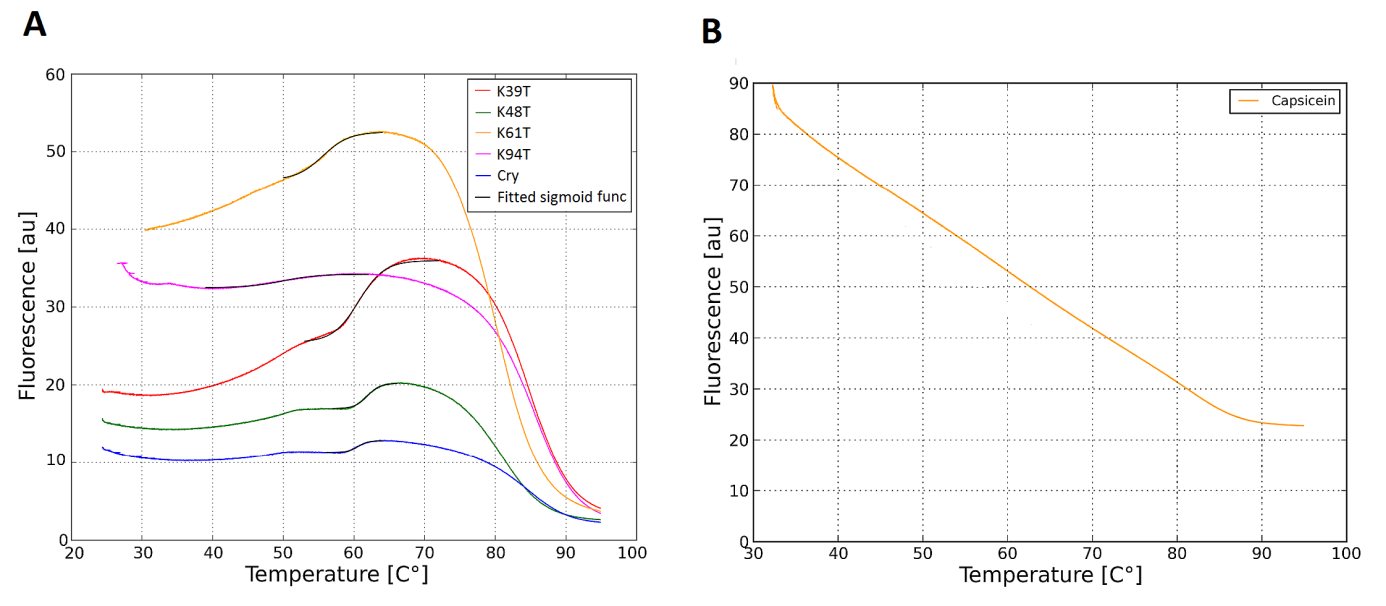


**Figure S2.**  *Thermal shift assay curves of cryptogein variants (A) and capsicein (B).* Thermal denaturation curves for the studied proteins measured on a LightCycler 480 instrument with 12.5x SYPRO Orange fluorescent dye. Individual proteins were measured in triplicate, corresponding melting temperature were determined by sigmoidal analysis of the raw fluorescence data (fitted region of the curve is shown in black).


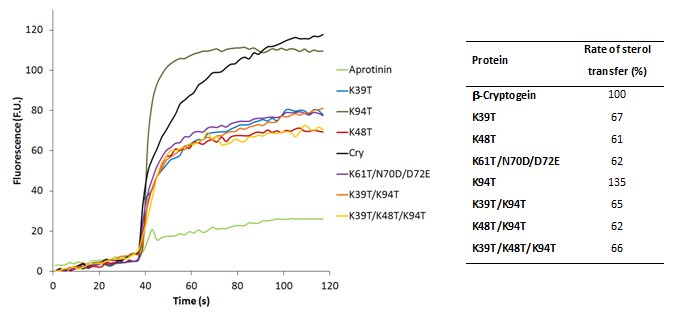


**Figure S3.** *Sterol transfer assay of cryptogein mutant forms*. The transfer of sterols between DHE and stigmasterol micelles catalyzed by cryptogein, its mutant variants and aprotinin, was measured by changes in the DHE fluorescence. Donor micelles contained DHE (0.63 µM), acceptor micelles contained stigmasterol (3 µM) in MES buffer. The transfer was induced by adding the studied proteins or aprotinin until DHE was equally distributed between donor and acceptor micelles. The rate of sterol transfer was measured from the increase in fluorescence after 2 min due to dilution of DHE in the stigmasterol micelles


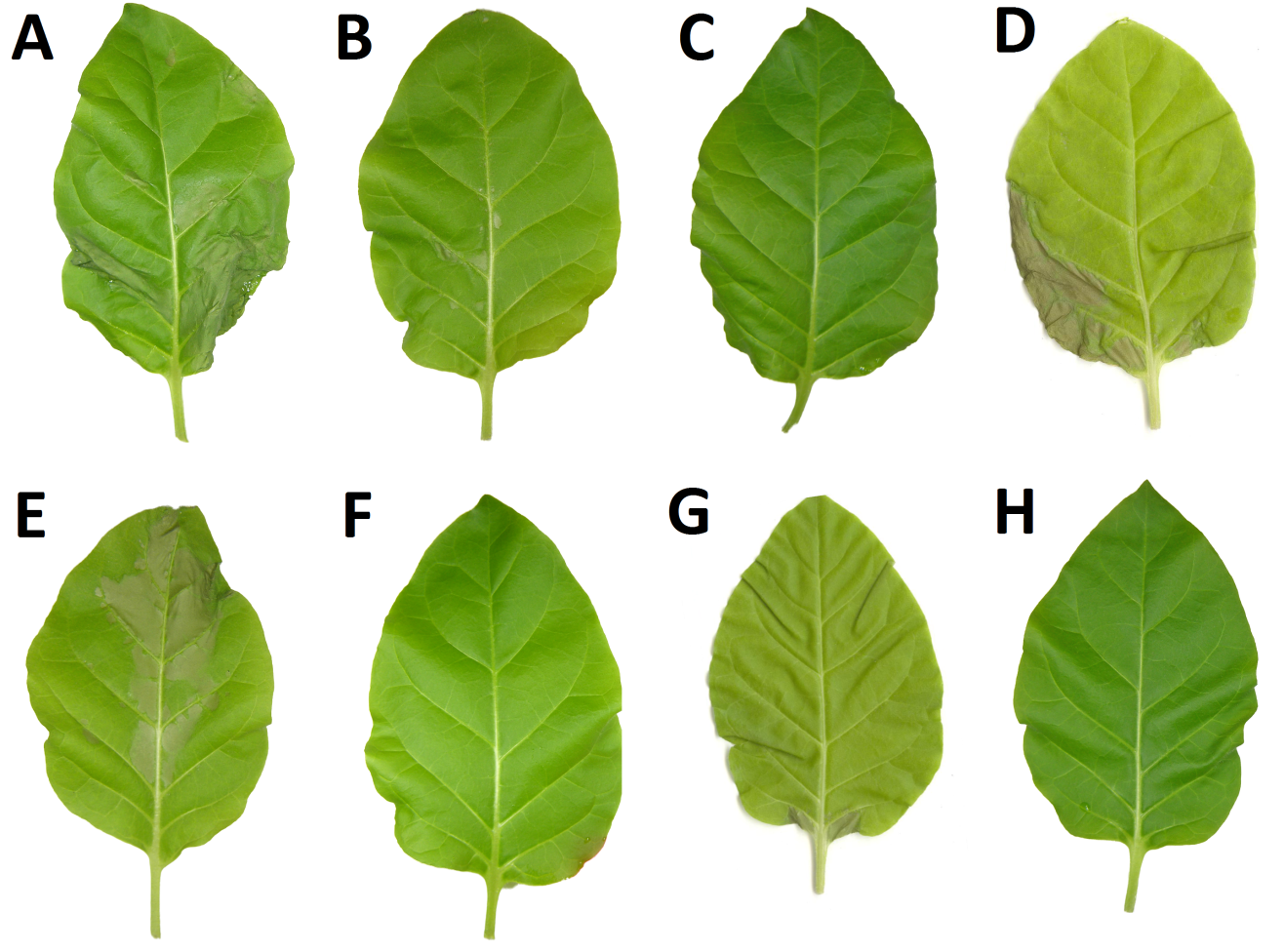


**Figure S4.** *Extent of leaf necrosis after petiole application of individual cryptogein variants.* (A) Leaves with the average extensive necrosis 3 days after application of 250 ng of cryptogein (A) and its mutant variants Lys39Thr (B), Lys48Thr (C), Lys61Thr/Asn70Asp/Asp72Glu (D), Lys94Thr (E), Lys39Thr/Lys94Thr (F), Lys48Thr/Lys94Thr (G) and Lys39Thr/ Lys48Thr/Lys94Thr (H).


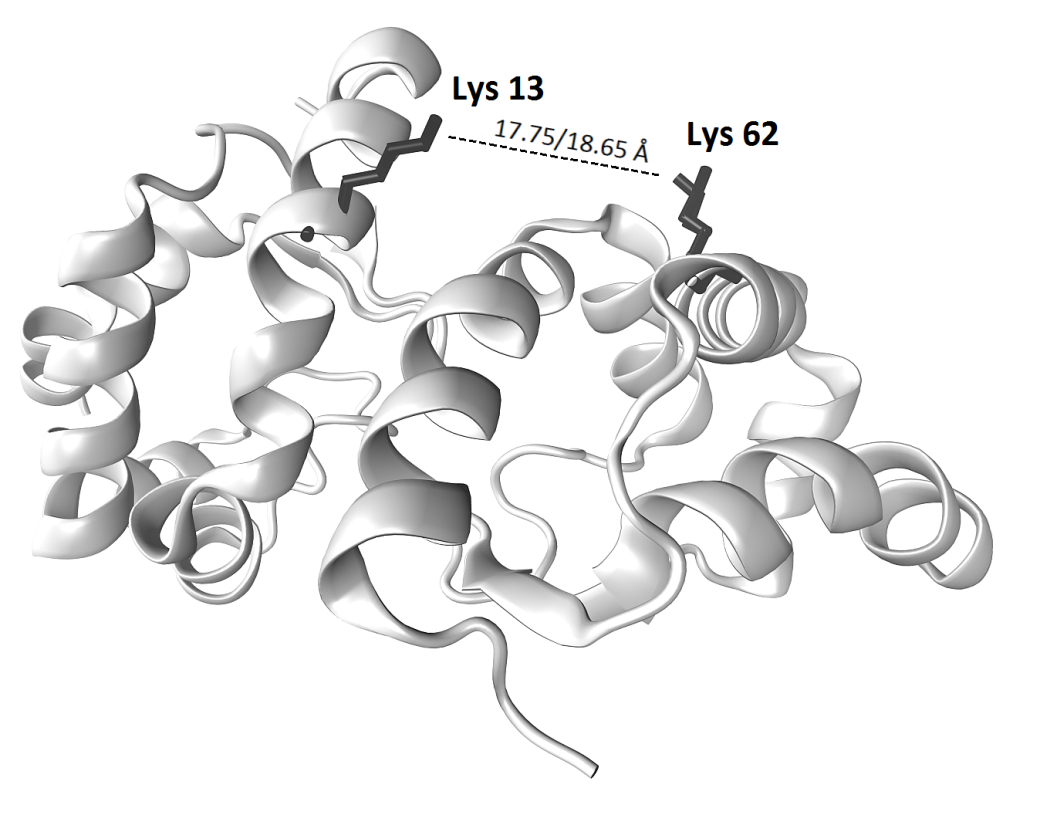


**Figure S5.** *Positions of the lysine residues 13 and 62 in a dimer structure of elicitin cinammomin.* The structure was visualized using the VMD program (Humphrey et al. 1996). Distance was calculated according to two possible locations of lysine residues in X-ray structure of beta-elicitin cinnamomin (PDB entry 2AIB).

***Measurement of tobacco nsLTP1 interaction with cryptogein***

*Interaction of cryptogein with non-specific Lipid Transfer Proteins 1 from tobacco*

To monitor potential interaction of nsLTP1 from tobacco (NCBI A.N. X62395) with cryptogein piezoelectric sensor was employed as well as in case of elicitin dimer formation measurement when previously well characterized nsLTP1 from tobacco (Buhot et al. 2004) was covalently attached to the crystal surface. Fitting of the measured decrease in frequency versus time to Eq. 2 using nonlinear regression yielded the value of *k*_obs_ for each concentration of cryptogein used. Plotting of these *k*_obs_ values against the molar concentration of cryptogein enabled a linear fit to the data (Figure S7), where the slope and intercept directly corresponded to the kinetic rate constants for association (*k*_a_) and dissociation (*k*_d_) (Eq. 3). The values of the corresponding kinetic equilibrium constants for nsLTP1-cryptogein formation were *K*_A =_ 5.61.10^5^ M^-1^ and *K*_D_ = 1.78.10^-6^ respectively.


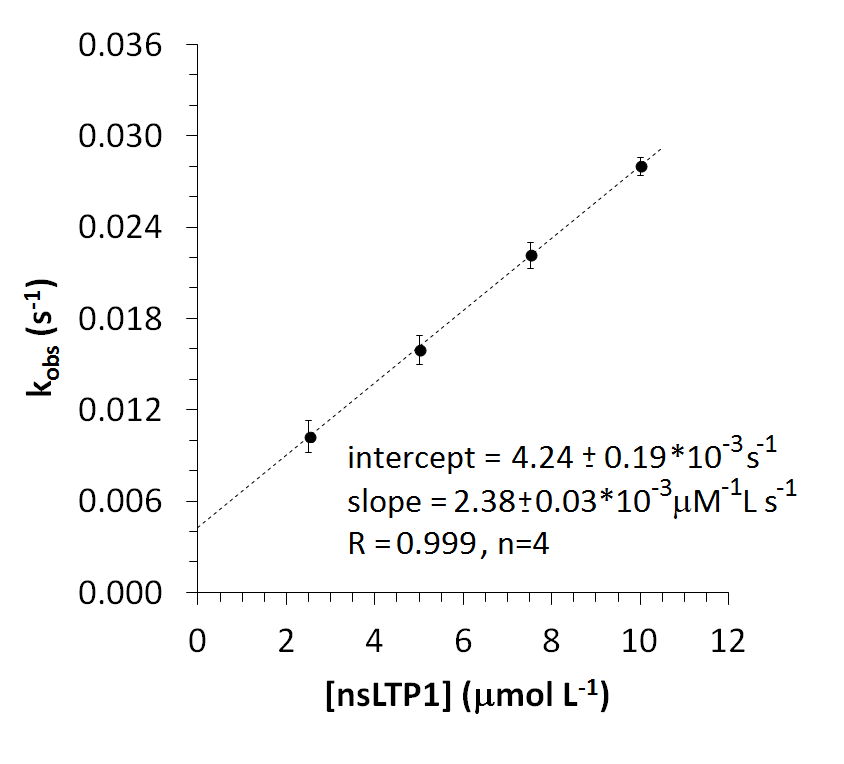


**Figure S6.** *Tobacco nsLTP1-cryptogein complex formation.* Plotting of calculated *k*_obs_ values against the molar concentration of proteins enabled a linear fit to the data (n=3), where the slope and intercept directly corresponded to the kinetic rate constants for association (*k*_a_) and dissociation (*k*_d_) of nsLTP1-cryptogein complex.

**REFERENCES**

**Humphrey, W., Dalke, A. and Schulten, K.** (1996). VMD: visual molecular dynamics. J Mol Graph 14, 33-8.

**Buhot N, Gomès E, Milat M-L, et al.** (2004) Modulation of the Biological Activity of a Tobacco LTP1 by Lipid Complexation. Mol Biol Cell 15(11), 5047-5052.
